# Publishing Chinese medicine knowledge as Linked Data on the Web

**DOI:** 10.1186/1749-8546-5-27

**Published:** 2010-07-27

**Authors:** Jun Zhao

**Affiliations:** 1 Image Bioinformatics Research Group, Department of Zoology, Oxford University, South Parks Road, Oxford, OX1 3PS, UK

## Abstract

**Background:**

Chinese medicine (CM) draws growing attention from Western healthcare practitioners and patients. However, the integration of CM knowledge and Western medicine (WM) has been hindered by a barrier of languages and cultures as well as a lack of scientific evidence for CM's efficacy and safety. In addition, most of CM knowledge published with relational database technology makes the integration of databases even more challenging.

**Methods:**

Linked Data approach was used in publishing CM knowledge. This approach was applied to publishing a CM linked dataset, namely RDF-TCM http://www.open-biomed.org.uk/rdf-tcm/ based on TCMGeneDIT, which provided association information about CM in English.

**Results:**

The Linked Data approach made CM knowledge accessible through standards-compliant interfaces to facilitate the bridging of CM and WM. The open and programmatically-accessible RDF-TCM facilitated the creation of new data mash-up and novel federated query applications.

**Conclusion:**

Publishing CM knowledge in Linked Data provides a point of departure for integration of CM databases.

## Background

Chinese medicine (CM) is yet to become an integral part of the standard healthcare system in Western countries due to a lack of scientific evidence for its efficacy and safety as well as a language and cultural barrier. This article presents a Linked Data approach to publishing CM knowledge in hope of bridging the gap between CM and Western medicine (WM).

The World Wide Web is a scalable platform for disseminating information through documents, having transformed how knowledge is learned and shared. Similarly, the Web may also be used as the platform for disseminating data. Linked Data [1] uses the Web as the information space to publish structured data rather than documents on the Web. In Linked Data, Uniform Resource Identifiers (URIs) are used to identify resources [2] and Resource Description Framework (RDF) is used to describe resources [3]. URIs are to data as what Uniform Resource Locators (URLs) are to web pages, providing identifications to resources; and RDF is to data as what HTML is to documents, providing descriptions about a resource in a machine-processable representation format.

Linked Data promises a new and more efficient paradigm for sharing and connecting distributed data, permitting decentralization and interoperability. Since Linked Data is built upon the Web Architecture [4], it inherits its decentralization and connectivity. The Web enforces no central control points and those distributed resources on the Web are intrinsically connected to each other by two fundamental elements, namely the Hyper-Text Transfer Protocol (HTTP) [5] which permits the transportation of information resources on the Web and the URIs which provide a globally-scoped system for identifying web resources (documents or data). Furthermore, linked datasets are meant to be interoperable based upon the Semantic Web standards established by the World Wide Web Consortium (W3C). These standards comprise RDF for publishing data in a structured format with explicit semantics and the SPARQL query language and protocol [6,7] for querying and accessing RDF data through an open and HTTP-based protocol.

A growing number of linked datasets as well as supporting tools and technologies are rapidly emerging, providing a unique opportunity for Linked Data to be applied in biomedical research and healthcare. The Linking Open Data (LOD) project [8] was founded in January 2007 and within one year the RDF published by the LOD community grew to over two billion [9]. The fast growth of Linked Data cloud cannot be achieved without the variety of open-source tools for publishing, searching, indexing and browsing linked datasets. Notably, tools such as D2R Server [10] and Triplify [11] are making relational databases accessible as RDF without transforming the source databases. Linked datasets become consumable for both humans and computers with the emergence of various Linked Data browsers such as Tabulator [12], Sig.ma [13], Linked Data query engines (e.g. SQUIN [14]) and Google-like Linked Data search engines (e.g. Sindice [15] and SWoogle [16]).

One of the earliest adopters of Linked Data for life sciences is the Bio2RDF project [17], in which various biological and bioinformatics knowledge bases have been published in the form of linked datasets using Semantic Web technologies. The knowledge bases published by Bio2RDF continue to grow, ranging from human genomics databases such as NCBI's Entrez Gene, proteiomics databases such as the Kyoto Encyclopedia of Genes and Genomes (KEGG) [18] and Protein Data Bank (PDB) [19] to pharmacogenomics databases such as PharmGKB [20], and cheminformatics databases such as PubChem [21]. Another active effort, similar to Bio2RDF, is the Linking Open Drug Data (LODD) project [22], founded under the umbrella of W3C Health Care and Life Science Interest Group. The goal of the LODD project is to gather requirements from the life science research community and to publish required databases in the Linked Data format. LODD has successfully published a selection of databases as Linked Data and generated their links with other Linked Data cloud [23], including the Bio2RDF datasets and the nucleus of Linked Data Cloud, namely DBpedia [24]. A missing link in the life science-oriented Linked Data cloud is a dataset about alternative medicines. Our RDF-TCM linked dataset plays a key role in connecting medical knowledge originating from different cultures and scientific disciplines. The aims of the presented article are as follows:

• Describing a CM linked dataset RDF-TCM, which is the first effort in publishing CM knowledge in a more accessible Linked Data format and is created according to our Linked Data Publication Methodology;

• Demonstrating that publishing linked CM data provides a point of departure for data integration through two efficient ways of consuming linked datasets.

## Methods

### TCMGeneDIT database

The RDF-TCM dataset transformed the relational TCMGeneDIT [25] as RDF. TCMGeneDIT not only provides information in English but also collects the associations among herbs, genes, diseases, CM effects and CM ingredients from public databases and literature. Existing knowledge is reused and some association information is collected through text mining techniques, such as:

• Herb names, such as *Ginkgo biloba*, were collected from the HULU TCM professional web site [26] and TCM-ID [27], a database on CM herbs and herbal ingredients;

• Ingredient data were collected from the above two resources as well as the Chinese medicine resource web [28];

• Human genes and their information were retrieved from NCBI Entrez [29];

• Disease names were extracted from the heading and entry term fields in the disease (C) section of the medical subject headings vocabulary (MeSH) [30];

• The relationship between genes and diseases were collected from PharmGKB [20];

• Many other association information between herbs and genes, diseases and effects were mined and extracted from a corpus of MEDLINE abstracts collected through PubMed.

### Create RDF-TCM

The TCMGeneDIT database is available as a database dump under the Creative Commons Attribution License [31]. To publish TCMGeneDIT as Linked Data, we followed our Linked Data Publication Methodology proposed previously [32], including the following steps:

1. Choose a transformation strategy, either through RDF caching or virtualization;

2. Design an URI scheme according to the Linked Data principles and the Cool URIs style [33], providing simple and stable URIs;

3. Construct schemas or ontologies based on the source data schemas, imposing as little interpretations as possible and reusing existing ontologies where possible;

4. Construct transformation scripts and mapping files, starting with transforming a small portion of the records and a test framework, which is not only useful for validating the sanity of the RDF dataset but also for revalidation when the transformation process is repeated;

5. Create mappings to other data sources where immediate values are foreseen, either using customized scripts or existing software tools such as Silk [34];

6. Finally, and preferably, provide metadata descriptions about the dataset, including its provenance information, and make all the scripts, configuration files, and ontologies accessible.

A skeleton of the methodology was proposed [32] and the following sections will provide details. Steps 2-5 should be applied iteratively and some design decisions must be made in accordance with fundamental principles.

#### Choose a transformation strategy

Linked datasets can be published either by creating RDF caching or through a virtualized access to the source data. RDF caching means that developers convert a snapshot of the source database into RDF and then load these cached data into an RDF store and publish it as Linked Data. The virtualization approach rewrites an HTTP-dereference request to a data URI into a query expressed in a language native to the source database (e.g. SQL) for evaluation against the data in their native form without transformation into RDF. The virtualization approach is more desirable if the source data have a high churn rate, but the performance of the current tools supporting this virtualization (such as Triplify [11]) is difficult to cope with large relational databases and complex rewriting rules. If the update rate of the source data is sufficiently low, the caching approach is more feasible. Because TCMGeneDIT is no longer updated, we chose the RDF caching approach to build RDF-TCM.

#### Design the URIs

URIs are required in Linked Data in order to identify entities (instances), types of entities (classes) and types of their relationships (properties). The 'Linked Data Principles' outlined by Berners-Lee [35] clarify the role of URIs in Linked Data and the set of best practices for publishing them:

"1. Use URIs as names for things; 2. Use HTTP URIs so that people can look up these names; 3. When someone looks up a URI, provide useful information using the standards (e.g. RDF, SPARQL); 4. Include links to other URIs, so that they can discover more things."

In addition we recommend that new URIs should only be coined if no existing URIs can be found and that they should be persistent. Reusing existing URIs improves the connectivity of a dataset with others and help establish shared names within the community. Consortia such as SharedNames [36] and Concept Web Alliance [37] are the active ongoing efforts in creating unique, shared names for biological entities. A data publisher should have control over the namespace under which new URIs are created, not only allowing useful information about these resources to be provided but also improving the stability of these URIs. Creating links to URIs published by others is highly recommended for bridging the gap between a local namespace and the Linked Data cloud.

The URIs used for RDF-TCM followed the pattern of:

http://purl.org/net/tcm/tcm.lifescience.ntu.edu.tw/id/{type}/{id}

where {type} corresponds to the type of an entity (such as Gene) and {id} is an identifier derived from the source data, e.g. the gene name or the herb name, or from a sequential number assigned by the transformation program. We used PURL [38] URIs to control the persistency of these URIs and we used the namespace of the TCMGeneDIT website as part of the URI to preserve some information about the owner and origin of the dataset. For example, the URI

http://purl.org/net/tcm/tcm.lifescience.ntu.edu.tw/id/medicine/Ginkgo_biloba

identifies the herb *Ginkgo biloba*.

And the URI

http://purl.org/net/tcm/tcm.lifescience.ntu.edu.tw/id/statistics/9199

denotes a statistics entity that describes confidence in the association relationship between some entities.

#### Design ontologies

Ontologies can be used as a controlled vocabulary to define the type of entities in a dataset and the type of relationships between them and to achieve a consistent interpretation about different datasets. A rich body of biological ontologies has been created and accumulated over the years [39]. When designing ontologies for describing linked datasets, we should reuse existing ontologies as much as possible. When a new ontology must be created, a conservative and incremental approach is recommended. Many of the linked datasets are published by a third party, rather than by the data provider. Documentation about these datasets is not always available. Imposing personal interpretations about the semantics of the data and its schema could introduce errors and should be avoided.

**Figure 1:**
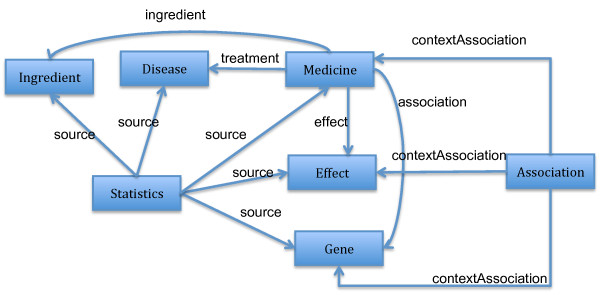
**The diagram of the RDF-TCM ontology**. The diagram illustrates the main classes (the boxes) and object properties (the directed arrows) in the RDF-TCM ontology http://purl.org/net/tcm-onto/. The data properties of the ontology are not shown.

As the data structure of TCMGeneDIT is very simple and there was no known TCM ontology by the time of creating the dataset, we created a simple CM ontology using OWL http://purl.org/net/tcm-onto/. The ontology contains seven classes, namely Gene, Medicine, Disease, Ingredient, Effect, Association and Statistics. Each entity of type Statistics describes statistics confidence in the associations between entities. Each entity of type Association represents an association between a Medicine, a Gene and a Disease. There are six object properties in total: five of them for relating a Medicine to a Gene, a Disease, its Ingredient, or its Effect and the last one, tcm:source, for pointing to the entities whose association relationship is described by a Statistics entity. There are five data properties whose domain is Statistics and whose value represents the statistics confidence in the association. For example, the value of tcm:medicine_effect_association_tvalue represents our confidence in the association between a Medicine and its Effect. A diagram capturing the structure of the ontology is shown in Figure 1. Note that the data properties associated with the Statistics class are not shown in the figure.

A Statistics entity was used to describe the statistical value of an association. Some associations relating to more than two entities such as the association relationship of medicine-gene-diseases cannot be expressed as RDF triples. To capture this n-ary relationship, we created Statistics entities to link together every entity involved in an association (see the example below) and to express the statistical value of the association using the data properties, e.g., tcm:medicine_effect_association_tvalue. The different types of data properties were created for different types of associations.

http://purl.org/net/tcm/tcm.lifescience.ntu.edu.tw/id/statistics/19087 a tcm:Statistics;

tcm:source

http://purl.org/net/tcm/tcm.lifescience.ntu.edu.tw/id/medicine/Acanthopanax_gracilistylus;

tcm:source http://purl.org/net/tcm/tcm.lifescience.ntu.edu.tw/id/disease/Retinoblastoma;

tcm:source http://purl.org/net/tcm/tcm.lifescience.ntu.edu.tw/id/gene/CDK2;

tcm:medicine_gene_disease_association_tvalue "1.414"^^xsd:float.

#### Data transformation

Data transformation should be incremental and test-driven. When transforming a new dataset into RDF or writing the configuration files for virtualization, developers should start with a small subset and avoid transforming the complete dataset. Loading a large number of RDF triples into an RDF store or retrieving very complex RDF descriptions for data entities by query rewriting can be a very time-consuming task and block the execution of following-on tests. A test framework should be designed forefront to spot any problems with the testing data and to ensure the sanity of the datasets, such as no blank nodes, no URIs containing invalid characters (e.g. space), no wrong property cardinalities, or no missing property values. These principles were applied when the relational TCMGeneDIT database was transformed into RDF.

#### Data linking

Links between datasets can be expressed with RDF. These links either reflect a type of relationship between entities or state a reconciliation between URIs published by various authorities. An example of the relationship type of links is to associate drugs from dataset *D*1 with genes from dataset *D*2 through a property such as *ex:targets*. Properties such as *owl:same*As or *rdfs:seeAlso *can be used for stating identity reconciliation. These RDF links allow users and Linked Data applications to start from one dataset and then follow on these RDF data links to move through a potentially endless web of data.

These data links can be created either during or after the creation of a linked dataset. Commonly, relating to another dataset (e.g., *ex:targets*) may be achieved as part of the transformation script, while mapping two URIs from different datasets may take place after a dataset is published and be executed either by their publishers or third parties.

The links may be created manually or automatically with open-source tools such as Silk [34]. However, identity reconciliation between biological entities is known to be difficult; string mapping is not always sufficient or reliable [40]. Developers should look for existing authoritative name mappings curated by data providers. Identifying the reference databases used by the source databases could help improve the precision of the mapping. For example, by understanding that the gene names used by TCMGeneDIT are from NCBI Entrez Gene for human, we can reduce the ambiguity of the mapping to the Entrez Gene dataset previously published by Neurocommons or Bio2RDF.

Extra attention should be given to any many-to-many mappings between URIs in the results. A manual cleaning of these mappings is highly recommended, requiring either the participation of domain experts or some contextual knowledge that are difficult to be expressed in computer programs.

The gene entities in the RDF-TCM dataset were linked with those from the NCBI Entrez Gene linked dataset [41] published by Neurocommons and those from the STITCH linked dataset [42] published by the Freie Universit*ä*t Berlin. Gene mapping was constructed with customized Python scripts based on the label of the genes. The mapping to Entrez Gene showed that 849 out of the total 945 RDF-TCM genes had a one-to-one mapping to an Entrez gene and that 95 of them had a many-to-many mapping to an Entrez gene and one of them was not mapped. The mapping to STITCH genes showed that 539 out of 943 mapped genes had a one-to-one mapping to a STITCH gene; and that 404 of them had a many-to-many mapping and two of them were not mapped. These many-to-many mappings were manually corrected so that only one-to-one mappings were in the results. We selected some sample data to manually confirm the correctness of the automatically generated one-to-one mappings. However, these automatic gene mappings were not thoroughly evaluated and this is an limitation of the work.

To link RDF-TCM with various other linked dataset from LODD, we used Silk, as part of the LODD project [23]. The mapping results by Silk have not been formally evaluated, but the correctness and completeness of Silk's approach were evaluated with other test datasets [34].

#### Data documentation

To improve the visibility of a dataset to Linked Data search engines such as Sindice, we recommend data publishers to describe their datasets using vocabularies such as the Vocabulary of Interlinked Datasets (voiD) [43] or the Provenance Vocabulary [44]. voiD is an RDF vocabulary for describing linked datasets on the Web in order to facilitate the discovery of these datasets and query federation applications. The Provenance Vocabulary is the first vocabulary to describe both the data creation and data access process related to a dataset on the Web.

A voiD file was published for RDF-TCM http://www.open-biomed.org.uk/void/rdf-tcm.ttl and the provenance of each RDF-TCM entity was described with the Provenance Vocabulary, published with Pubby [45], a Linked Data publication tool extended with a provenance component. We published all our Python scripts for transforming the database dump into RDF and for linking RDF-TCM to other datasets. All the scripts can be found at http://code.google.com/p/junsbriefcase/source/browse/#svn/trunk/biordf2009_query_federation_case/tcm-data.

## Results

### RDF-TCM dataset

The RDF-TCM dataset contained 111,021 RDF triples, providing association information for 848 herbs, 1064 ingredients, 241 putative effects, 553 diseases and 945 genes. This dataset was linked with a variety of life science linked dataset including:

• Entrez Gene dataset, part of the HCLS knowledge base, derived from the NCBI Entrez Gene database

• DrugBank http://www4.wiwiss.fu-berlin.de/drugbank/: derived from DrugBank [46] published by the University of Alberta, containing detailed information about almost 5,000 FDA-approved small molecule and biotech drugs

• DailyMed http://www4.wiwiss.fu-berlin.de/dailymed/: derived from Dailymed [47] published by National Library of Medicine (NLM), containing high quality packaging information on 4,300 marketed drugs

• SIDER http://www4.wiwiss.fu-berlin.de/sider/: derived from SIDER database [48] published by EMBL Germany, containing side effect information on 930 marketed drugs

• Diseasome http://www4.wiwiss.fu-berlin.de/diseasome/: derived from the Diseasome dataset [49] which publishes a network of disorders and disorder genes, obtained from Online Mendelian Inheritance in Man (OMIM)

• STITCH http://www4.wiwiss.fu-berlin.de/stitch/: derived from STITCH [50] published by EMBL Germany, containing information about known or predicted interactions between proteins and chemicals

• PharmGKB http://bio2rdf.org/ published by Bio2RDF: derived from PharmGKB [51] published by Stanford University, sharing knowledge about the impact of human genetic variations on drug response and publishing data, among many others, about the associations between drugs, genes and diseases curated by domain experts

Table 1 summarizes the type of entities that link RDF-TCM with each of the above dataset and the number of each type of links. All these link datasets can be downloaded as RDF dumps http://purl.org/net/tcmdata/ or accessed through the public SPARQL endpoint http://www.open-biomed.org.uk/sparql/. In the following section, we will demonstrate how this RDF dataset and these RDF links data are used to assist the exploitation of CM and WM.

**Table 1:** A summary of different types of links between RDF-TCM and other datasets

Dataset	Type of linked entities	Properties used for interlinking	Number of links
Entrez gene	Genes	Symbols of the genes	944
Diseasesome	Diseases	Labels of the disease names	63
	Genes	Symbols of the genes	312
SIDER	Diseases	Labels of the disease names	171
Drugbank	Genes	Symbols of the genes	384
Dailymed	Ingredients	Labels of the ingredient names	21
	Genes	Symbols of the genes	649
DBpedia	Diseases	Labels of the disease names	255
	Herbs	Labels of the herb names	438
STITCH	Genes (encoding proteins)	Names of the genes	937
PharmGKB	Genes	Names of the genes	202

### Search for potential alternative medicines by mash-ups

**Figure 2:**
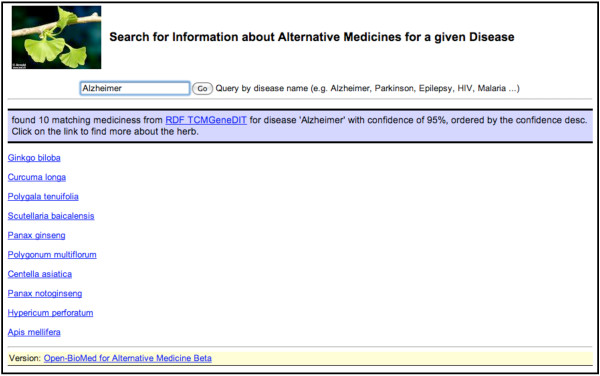
**The data mash-up application for alternative medicines**. A search for alternative medicines for the Alzheimer's disease takes a disease name as the input and search in the RDF-TCM dataset for a list of possible alternative medicine associated with the disease.

**Figure 3:**
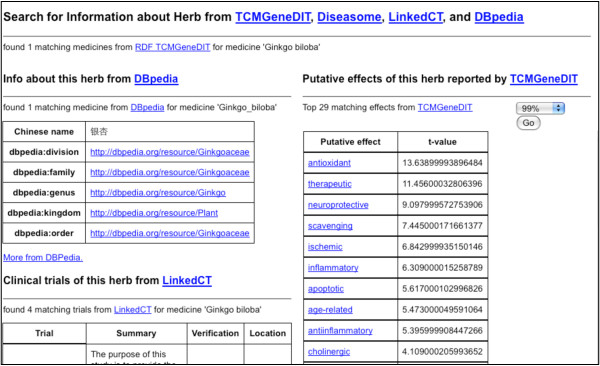
**Detailed information about each alternative medicine**. More information about *Ginkgo biloba *is returned, including its general information retrieved from DBpedia (left-side pane) and its putative effects information retrieved from RDF-TCM (right-side pane). This query demonstrates how we can create a more complete picture of knowledge about *Ginkgo biloba *by querying distributed linked datasets.

**Figure 4:**
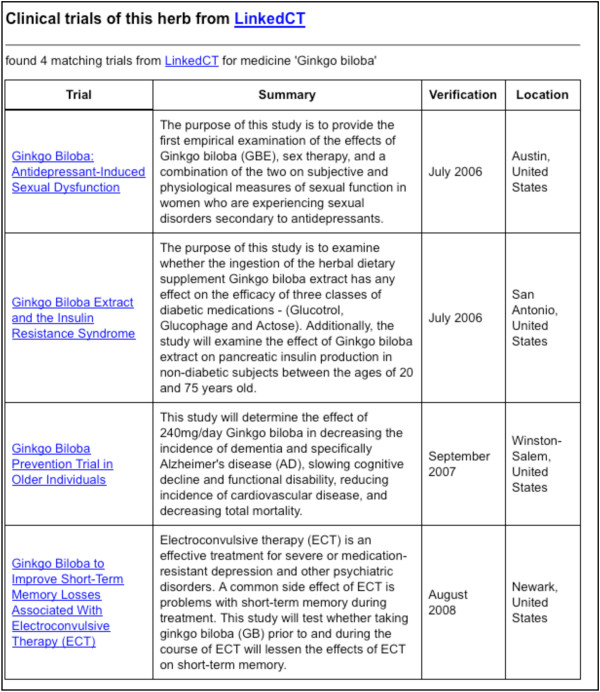
**Clinical trials related to Ginkgo biloba**. Clinical trials related to *Ginkgo biloba *are found from the LinkedCT dataset. These results are also linked to LinkedCT where more information about these trials can be found.

**Figure 5:**
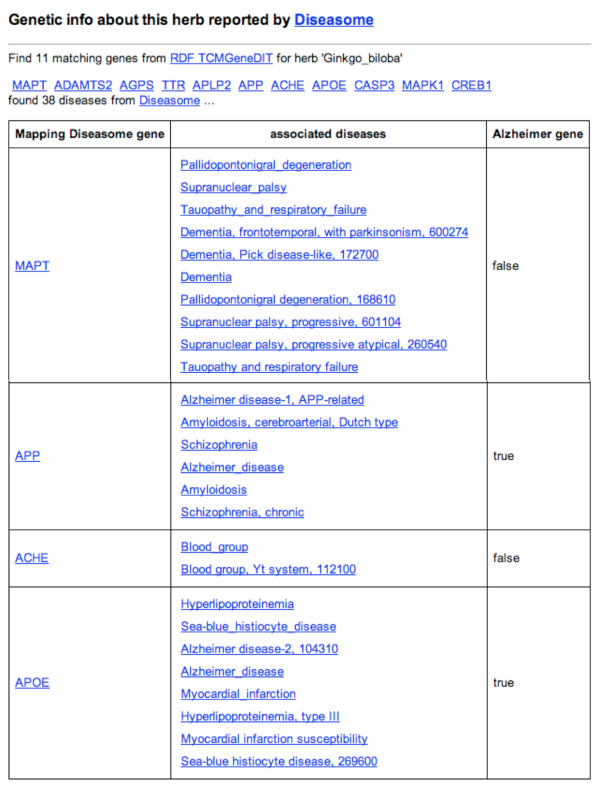
**Confirmation of genetic evidences for the efficacy of alternative medicines using RDF-TCM and Diseasome**. We first use the RDF-TCM dataset to find genes associated with the Alzheimer's diseases and the herb *Ginkgo biloba*, and we then use the Diseasome database to search for the diseases associated with these genes. If an RDF-TCM gene is also associated with the Alzheimer's disease according to Diseasome, we then confirm that gene as an Alzheimer's gene. In this way, we use two datasets created by two different medical research communities to confirm genetic evidence for the herbs.

Here we present an application [52] of the RDF-TCM dataset as an example. As shown in Figure 2, the data mash-up application allows users to first search for alternative medicines for a diseases using the disease and herb association information from RDF-TCM. The result was ranked by the statistical value from the TCMGeneDIT database that states the confidence in the association between diseases and herbs, i.e. Ginkgo biloba has the highest score for its association with the Alzheimer's Disease. Users may then retrieve detailed information about each alternative medicine (Figure 3, 4 and 5). The scientific classification information was retrieved from DBPedia and putative effects of herbs were retrieved from RDF-TCM (Figure 3). Related clinical trial information were retrieved from the LinkedCT dataset (Figure 4) hosted by the EU LarKC project [53] with string matching SPARQL queries. Figure 5 shows how this application may also help confirm the association relationship between a herb, its possible disease targets and the genes affected by these diseases by combining the WM knowledge from Diseasome and RDF-TCM. The application is an Ajax application implemented with Javascript. Each widget in the application executed a SPARQL query to one or multiple SPARQL endpoints and presented the query result in the web browser in a user-friendly way. The application requires that a data source must be accessible through a SPARQL endpoint. This data mash-up application bridged the knowledge connection between CM and WM. Instead of making users browse various possible data sources to gather information about herbs, the mash-up provides a central point for searching for knowledge about CM gathered from various sources published by these two scientific communities.

### Search for potential alternative medicines by the Linked Data approach

**Figure 6:**
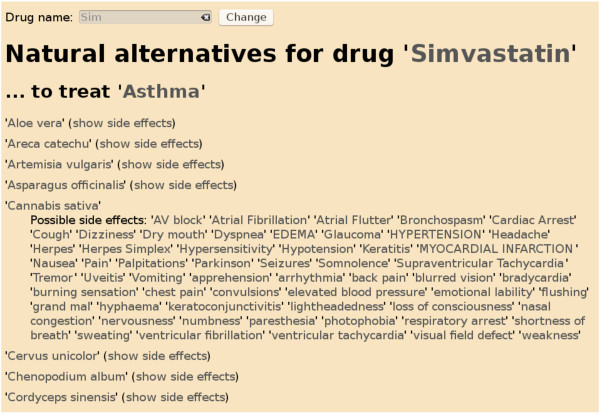
**Finding alternative medicines as well as their side effects powered by SQUIN**. To find alternative medicines to *Simvastatin *as well as their side effects powered by SQUIN, we use a Linked Data query engine, which allows one SPARQL query to access 6 distributed linked datasets published at different sources, including Drugbank, Diseasome, SIDER, LinkedCT, Dailymed and RDF-TCM.

RDF-TCM together with LODD forms a web of medical data, accessible through Linked Data query engines as a single dataspace. SQUIN [14] is one such Linked Data query engine that traverses the whole Web of Data to retrieve all relevant data sources for a query by taking the URIs in the query or in the intermediate results and following links of these URIs to other data sources. In this second application [54], to search for an alternative medicine to a Western medicine (Figure 6) we used SQUIN to take the example SPARQL query in Listing 1 to traverse 7 distributed Linked Datasets including Drugbank, Diseasome, SIDER, LinkedCT, Dailymed and RDF-TCM.

Listing 1: The SPARQL query for finding alternative medicines to Simvastatin.

PREFIX tcm: http://purl.org/net/tcm/tcm.lifescience.ntu.edu.tw/

PREFIX drugbank: http://www4.wiwiss.fu-berlin.de/drugbank/resource/drugs/

PREFIX rdfs: http://www.w3.org/2000/01/rdf-schema#

PREFIX owl: http://www.w3.org/2002/07/owl#

PREFIX rdf: http://www.w3.org/1999/02/22-rdf-syntax-ns#

SELECT DISTINCT ? diseaseLabel ? altMedicineLabel

WHERE {

http://www4.wiwiss.fu-berlin.de/drugbank/resource/drugs/DB01273

drugbank: possibleDiseaseTarget ? disease.

? disease owl: sameAs ? sameDisease.

? altMedicine tcm: treatment ? sameDisease.

? altMedicine rdf: type tcm: Medicine.

? sameDisease rdfs: label ? diseaseLabel.

? altMedicine rdfs: label ? altMedicineLabel.

}

## Discussion

The data mashups and the SQUIN-powered application demonstrate how Linked Data may serve as the point of departure for data integration. It allows developers to access machine-processable datasets either using the exible SPARQL query language or using Linked Data query engines (e.g. SQUIN) to access distributed information as one Web of Data. These two different approaches are complementary: the SQUIN-powered application may be included as one of the widgets in the mash-up application, and the mash-up approach may be used to support applications that need to perform schema and semantic mappings between datasets, which cannot be achieved with SQUIN.

Publishing RDF-TCM as Linked Data enables us to address some disadvantages of data integration approaches based on the relational database technologies [55], which are not necessarily unique to CM data resources. Firstly, Linked Data helps us address the identity linking and management. Most relational life science databases tend to use a local identifier for their data resources, even though overlapping information or existing identifiers have been provided elsewhere. Integrating these databases must first overcome the identity mapping problem. Linked Data promotes the use of uniform resource identifiers, i.e the URIs. Although uniform identifiers are yet to be established, there are ongoing active efforts in drawing together the community. Moreover, Linked Data allows the interlinking between URIs to be expressed in structured and explicit statements, such as RDF statements. Such RDF data links may be published by anyone and kept independent of the datasets. The other issue related to relational database integration is that often no programmatic access is provided for these databases and only a data dump is available. Linked Data on the other hand enables descriptions about an entity to be expressed in structured format (i.e. RDF) and retrievable by its URI. Linked Data also allows datasets to be accessible through the standard SPARQL query language and protocol. Our example applications have demonstrated how these two ways of consuming RDF-TCM provide the flexibility of integrating biomedical knowledge available in Linked Data format.

In contrast to the existing ontology-based approach [56,57], our RDF-TCM dataset is described with a very lightweight schema to publish a large number of instances. Associating lightweight semantics reduces the cost in publishing data and such datasets can satisfy most initial user requirements; while the heavier semantic approach would require more efforts in ontology engineering that makes data publication much more expensive. Linked data is most useful to data integration tasks at a syntactic level, such as the two example applications presented here; an ontology-based approach would be more useful for addressing requirements and issues requiring a controlled vocabulary to link together information at the semantic level. Investigating whether the latter approach would be needed for a Linked Data approach, such as one providing the integration of medical datasets by the disease names (and their classifications), is part of our future work.

## Conclusion

The Linked Data approach provides a set of best practices encouraging data providers to publish their data in an openly-accessible and programmatically-accessible manner. The benefit of such approach is demonstrated by the two examples in this study, consuming linked datasets to build useful applications. As improved tools and technologies of Linked Data are being made available, the CM and WM linked datasets will increase in number and volume through stepwise changes in multilingual publication and query practices among the CM community and become openly accessible to a larger community. Our Linked Data publication methodology reduces the efforts and errors in publishing linked datasets by systematizing and explicating the design decisions. Our further work is the evaluation of the correctness and completeness of the mapping between different datasets.

## Abbreviations

CM: Chinese Medicine; WM: Western medicine; URIS: Uniform Resource Identifiers; RDF: Resource Description Framework; URLS: Uniform Resource Locators; HTTP: Hyper-Text Transfer Protocol; W3C: World Wide Web Consortium; LOD: Linking Open Data; KEGG: Kyoto Encyclopedia of Genes and Genomes; PDB: Protein Data Bank; LODD: Linking Open Drug Data; MESH: Medical Subject Headings Vocabulary; VOID: Vocabulary of Interlinked Datasets; NLM: National Library of Medicine; OMIM: Online Mendelian Inheritance in Man.

## Competing interests

The author declares that they have no competing interests.

## Authors' contributions

The author conducted the research and wrote this article.
